# Australian podiatrists scheduled medicine prescribing practices and barriers and facilitators to endorsement: a cross-sectional survey

**DOI:** 10.1186/s13047-022-00515-w

**Published:** 2022-02-08

**Authors:** Kristin Graham, Lisa Matricciani, Helen Banwell, Saravana Kumar, Ryan Causby, Saraid Martin, Lisa Nissen

**Affiliations:** 1 Allied Health & Human Performance, The University of South Australia, North Terrace, Adelaide, SA 5000 Australia; 2 Clinical & Health Sciences, The University of South Australia, North Terrace, Adelaide, SA 5000 Australia; 3 Faculty of Health School of Clinical Sciences, Queensland University of Technology, Brisbane, QL 4000 Australia

**Keywords:** Podiatry, Prescribing, Endorsement for scheduled medicine

## Abstract

**Background:**

Non-medical prescribing is one healthcare reform strategy that has the potential to create health system savings and offer equitable and timely access to scheduled medicines. Podiatrists are well positioned to create health system efficiencies through prescribing, however, only a small proportion of Australian podiatrists are endorsed to prescribe scheduled medicines. Since scheduled medicines prescribed by Australian podiatrists are not subsidised by the Government, there is a lack of data available on the prescribing practices of Australian podiatrists. The aim of this research was to investigate the prescribing practices among Australian podiatrists and to explore barriers and facilitators that influence participation in endorsement.

**Methods:**

Participants in this quantitative, cross-sectional study were registered and practicing Australian podiatrists who were recruited through a combination of professional networks, social media, and personal contacts. Respondents were invited to complete a customised self-reported online survey, developed using previously published research, research team’s expertise, and was piloted with podiatrists. The survey contained three sections: demographic data including clinical experience, questions pertaining to prescribing practices, and barriers and facilitators of the endorsement pathway.

**Results:**

Respondents (*n* = 225) were predominantly female, aged 25–45, working in the private sector. Approximately one quarter were endorsed (15%) or in training to become endorsed (11%). Of the 168 non-endorsed respondents, 66% reported that they would like to undertake training to become an endorsed prescriber.

The most common indications reported for prescribing or recommending medications include nail surgery (71%), foot infections 474 (88%), post-operative pain (67%), and mycosis (95%). The most recommended Schedule 2 medications were ibuprofen, paracetamol, and topical terbinafine. The most prescribed Schedule 4 medicines among endorsed podiatrists included lignocaine (84%), cephalexin (68%), flucloxacillin (68%), and amoxicillin with clavulanic acid (61%).

**Conclusion:**

Podiatrists predominantly prescribe scheduled medicines to assist pain, inflammatory, or infectious conditions. Only a small proportion of scheduled medicines available for prescription by podiatrists with endorsed status were reportedly prescribed. Many barriers exist in the current endorsement for podiatrists, particularly related to training processes, including mentor access and supervised practice opportunities. Suggestions to address these barriers require targeted enabling strategies.

**Supplementary Information:**

The online version contains supplementary material available at 10.1186/s13047-022-00515-w.

## Background

In Australia there is a documented shortage of medical practitioners in many areas which impacts on the prescription of scheduled medicines amongst other concerns [1]. This shortage will only increase in the context of increases in life expectancy, rising chronic disease, and rural and remote workforce shortages [2–4]. Non-medical prescribing is one healthcare reform strategy that has the potential to offer equitable and timely access to scheduled medicines [2, 5], and allied health professionals have been identified internationally as of high value in this role [6]. The increasing use of non-medical prescribing has been shown to be cost effective and to improve patient satisfaction while not compromising care or safety [7–9]. Such strategies aim to build a flexible, responsive, and sustainable Australian healthcare workforce that fully utilises workforce resources and competencies [3, 4, 10].

Podiatrists frequently assess, diagnosis, and intervene in painful musculoskeletal injuries, inflammatory conditions, skin and soft-tissue infections, high risk and diabetic foot disease, and fungal infections [11]. Therefore, endorsed podiatrists are well positioned to reduce inconveniences and prevent duplicate visits to general practitioners. Timely prescription of scheduled medicines, such as antibiotic therapy for infected foot ulcers, may prevent deterioration and subsequent hospitalisation and/or limb amputation. Similarly, the ability to prescribe appropriate analgesia and anti-inflammatory medications for acute musculoskeletal injuries and inflammatory condition (such as gout) may reduce complications and emergency department presentations.

In Australia medicines and poisons are classified by schedules which correspond to the level of regulatory control over their availability, which are published in the Standard for the Uniform Scheduling of Medicines and Poisons (SUSMP) [12]. The schedules relevant to the National Podiatry Scheduled Medicines List include Schedule 2 (S2) which are substance that require advice from a pharmacist or licenced person for safe use, Schedule 3 (S3) which require professional advice for safe use but do not require a script, Schedule 4 (S4) which are prescription only medicines, and Schedule 8 (S8) which are controlled drugs [13]. Australian podiatrists with general registration, may administer, obtain, possess, sell, supply, or use (recommend) a range of S2 and S3 substances for the treatment of podiatric conditions, and may be authorised to possess and administer local anaesthetic agents as per the state and territory legislation in which they practice [14].

The Health Practitioner Regulation National Law, enacted by all Australian States and Territories, enables the national health practitioner boards to endorse the registration of suitably qualified health practitioners to prescribe scheduled medicines (endordsed) [13]. The minimum requirements established by the Podiatry Board of Australia to gain endorsement include holding an approved qualification (Pathway A) or completing a portfolio of evidence that includes a supervised practice component (Pathway B). This registration allows endorsed podiatrists to administer, obtain, possess, prescribe, sell, supply or use (prescribe) a broader range of medicines including S4 and S8 medicines from the National Podiatry Scheduled Medicines List, for the treatment of podiatric conditions [13]. Importantly, in addition to national regulation, podiatrists must comply with the legislation and regulations of the State and Territory in which they practice [13]. Therefore, the schedule medicines podiatrist can administer, obtain, possess, prescribe, sell, supply, or use may vary across jurisdictions. Although the Podiatry Board of Australia has been able to endorse podiatrists to prescribe scheduled medicines since 2010, less than 3% of Australian registered podiatrists have gained endorsement [15].

Podiatrists are not covered under the Australian Pharmaceutical Benefits Scheme (PBS) [16], which provides medicines at a Government-subsidised price and collates prescription histories. Therefore, there is a lack of quantitative information about prescribing practices of podiatrists, making it difficult to assess if endorsement for podiatrists leads to health system efficiencies or improved patient access and outcomes.

Little is known about why the uptake for endorsement for scheduled medicines remains low among Australian podiatrists. Graham and colleagues [17], in a qualitative thematic analysis of 13 podiatrists with and without endorsement, identified competence and autonomy (i.e. need/desire to broaden current scope of practice); social and workplace influences (i.e. access to mentors, supervised practice opportunities), and extrinsic motivators (i.e. time and cost of becoming in endorsed) are key barriers and facilitators for podiatrists gaining endorsement. These factors are yet to be explored within the broader podiatry population.

The aim of this research was therefore to investigate medicines prescribing and recommendation practices among Australian podiatrists and, to explore barriers and facilitators that influence the uptake of endorsement.

## Methods

### Research design

This research was a quantitative, cross-sectional survey design, conducted between July 2020 and December 2020. The online survey was created and delivered via SurveyMonkey®. Potential participants were provided with written information on the study aims and directives, giving online informed consent prior to commencing the survey. Respondents were advised that they could withdraw from the survey at any time by closing the browser, with data collected to that point included in the results. Those completing the survey were offered the option to provide an email address if they would like a summary of results supplied, otherwise all survey respondents remained anonymous. Ethical approval was gained from the University of South Australia Human Research Ethics Committee (Approval number 202938).

### Participants and settings

All registered and practicing Australian podiatrists were eligible to participate (*n* = 5759) [15]. Participants were alerted to, and invited to participate in, the research via the Australian Podiatry Association, Facebook™, Twitter™, special interest groups, and through the authors’ networks. This approach was particularly chosen to ensure maximum coverage of podiatrists, promote participation and achieve adequate response rate.

### Survey design

Data were collected during a single round, purpose built, self-reported survey. The survey was developed collaboratively by the author group and pilot tested by three podiatrists, who were then excluded from the results, to ensure clarity and appropriateness of question structure, as well as face validity. The survey contained three sections: demographic data including clinical experience, questions pertaining to medicines prescribing or recommendation practices, and perceptions of the endorsement pathway.

Demographic data included registration type (podiatrist or podiatric surgeon), gender, age, state, or territory of most frequent practice, primary role (clinician, administrator, teacher, or educator, researcher), primary work sector (private, public, or both private and public), employment status (self-employed, employed etc.,), and location. Participants then identified as non-endorsed, endorsed, or undertaking the requirements for Pathway B to become endorsed for prescribing scheduled medicines (in-training), the location of practice where they were most likely to prescribe medications, and length of time since endorsement where relevant.

Questions pertaining to medicines prescribing and recommendation practices asked participants to identify how often (weekly, monthly, quarterly, annually, never) they prescribed (or recommended) from the medications listed on the Podiatry Board of Australia: Guidelines for endorsement for scheduled medicines [10]. The medicines were grouped into: antimycotics, antibacterial, actinic keratosis, drugs for gout, corticosteroid, non-steroidal anti-inflammatories, analgesics, antihistamines, antidotes and antivenoms, local anaesthetics, emergency (anaphylactic reactions), and benzodiazepines. If participants had prescribed or recommended from a medication group, a drop-down list asked the specific medicaments prescribed from those approved for use by Australian podiatrists [13]. For a full list of responder choices, please see Supplementary Material Table 1.

**Table 1:** Demographic details of participants and comparisons with all registered Australian podiatrists

Demographics	AHPRA registrant data	Participants Endorsement status, ***n*** = 225		
		Non-endorsed	Endorsed	In-training
Total registered, n (endorsed)	5783 (162)	168	33	24
General registration	5604	168	29	24
Podiatric surgeon	36		4	
**Sex**				
Male	2370	52	15	8
Female	3413	116	18	15
**Age**				
< 25	344	14	0	4
25–29	1204	31	2	5
30–34	1104	29	8	6
35–39	793	29	7	2
40–44	613	21	1	1
45–49	587	15	1	4
50–54	459	13	5	2
55–59	347	11	6	0
> 60	223	5	3	0
**State, n (endorsed)**				
ACT	76 (2)	1	0	0
NSW	1631 (11)	29	2	0
NT	30 (1)	1	0	1
QLD	999 (41)	18	11	0
SA	534 (18)	60	7	5
TAS	119 (4)	1	0	2
VIC	1818 (49)	55	12	8
WA	512 (31)	3	1	8
**Work sector**				
Public and Private		12	4	1
Private sector-employee		44	4	7
Private-other		5	1	0
Private-self-employed		67	15	8
Public sector		40	9	10
Location*				
Major Cities of Australia		108	25	15
Inner regional Centre		25	4	6
Outer regional Centre		24	4	3
Remote		10	0	0
Very remote community		1	0	0
**Years of practice**				
0–4		42	1	13
5–9		27	6	2
10–14		32	10	3
15–19		24	2	
> 20		43	14	21
**Years of endorsement**				
< 1 year		n/a	4	n/a
1–4 years		n/a	10	n/a
5–9 years		n/a	10	n/a
10–15 years		n/a	4	n/a
**Setting most often prescribe**				
Private practice		n/a	15	n/a
Community health care service		n/a	1	n/a
Outpatient service		n/a	6	n/a
Aged care		n/a	0	n/a
Hospital		n/a	4	n/a
Education facility		n/a	1	n/a
Sports center		n/a	0	n/a
Locum private practice		n/a	0	n/a
Aboriginal health service		n/a	0	n/a

*Location was defined using the Australian Statistical Geography Standard (ASGS), which defines relative remoteness, using the Accessibility and Remoteness Index of Australia (ARIA+). Further detail has been reported elsewhere [18].

Note: In-training = Undertaking the process to gain endorsement

Questions concerning barriers and facilitators to endorsement were developed based on the previous qualitative study conducted by Graham and colleagues [17]. Specifically, endorsed and in-training participants were asked to identify which facilitators, from a given list, most contributed to their decision to undertake endorsement. Example facilitators were ‘It would enable me to offer complete patient care’, ‘I believe it is an essential skill for effective podiatry practice’. This same group then identified items that made it difficult to complete the requirements for endorsement. Examples include ‘The time commitment involved impacted my private life’ and ‘Limited access to supervisors/mentors’.

### Procedure

Participants were asked to indicate their endorsed status (non-endorsed, in-training, or endorsed), and the survey tool used skip-logic to skip to relevant questions. All responders were asked the same questions regarding demographics, prescription/recommendation practices and facilitators for endorsement, however, endorsed or in-training podiatrists were asked some additional questions specific to barriers surrounding the endorsement procedure.

### Data management

Participants were categorised by endorsement status for descriptive purposes (e.g., Non-endorsed, Endorsed, and In-training). For questions relating to barriers and facilitators to training (i.e., survey section three), In-training and Endorsed outcomes are pooled as both groups have insight into undertaking the process to gain endorsement.

### Data analysis

Data collected were de-identified and exported to Microsoft Excel (Microsoft Corporation (2018)) for descriptive analysis. All responses were presented as reported, except for the length of time respondents have held endorsements. For this question, responses were analysed as five categories of duration (< 1, 1–4, 5–9, 10–15 years) to align with durations reported by Australian Health Practitioner Regulation Agency (AHPRA) registrant summary report. Results are presented as frequencies.

## Results

Of the 229 participants who agreed to take part in the survey, four failed to report their profession and were excluded from all further analyses. A total of 225 registered Australian podiatrists were included in the results for this survey, four of whom were podiatric surgeons.

### Demographic information & clinical experience of respondents

Descriptive data of registered podiatrists who took part in the survey are presented in Table 1. Respondents were predominantly female, aged 25–45, working in the private sector. Most endorsed prescribers practiced in Queensland (33%) or Victoria (36%), worked within the metropolitan regions of Australia (86%) in private practice (73%), with over 10 years of clinical experience (79%). Most endorsed prescribers had less than 10 years of endorsement (86%) and none of the participants who reported that they were endorsed prescribers or undertaking the process to gain endorsement worked in remote or very remote parts of Australia. As presented in Table 1, the survey participants reflect the AHPRA registrant data published in March 2021 [15].

Of the 225 participants who held general registration, approximately one quarter (25.3%) were endorsed (*n* = 33, 14.7%) or undertaking the process to become endorsed (*n* = 24, 10.7%) (this included those completed and awaiting board approval). All podiatric surgeons (*n* = 4) were endorsed. Of the 168 non-endorsed respondents, 66.0% reported that they would like to gain endorsement.

### Prescribing practices

**Fig. 1:**
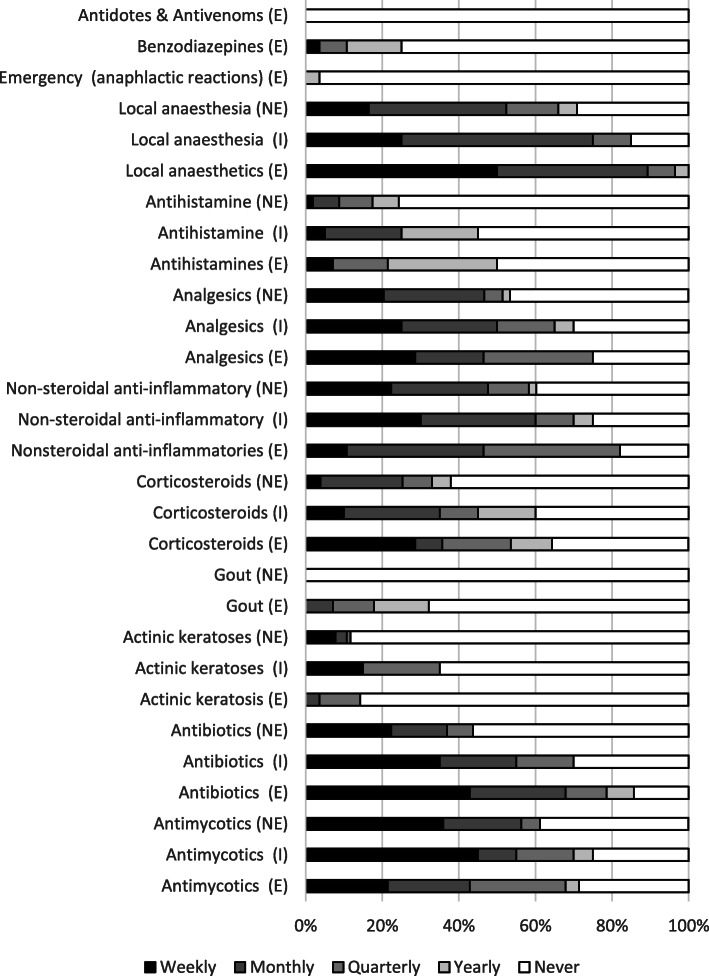
Frequency of medication class prescribed/recommended over the last twelve months according to endorsement status Note: (E) Endorsed (NE) Non-endorsed (I) In-training (undertaking the process to gain endorsement)

The frequency of medications prescribed or recommended over the last 12-month period (weekly, monthly, quarterly, yearly, never) according to endorsement status are presented in Fig. 1. The groups of medications most frequently prescribed or recommended (on a weekly or monthly basis) by all respondents include local anaesthetics, antibacterial agents, analgesia, antimycotics, and non-steroidal anti-inflammatory drugs. The most common indications reported for prescribing/recommending these medications include nail surgery (71%), foot infections and ulcerations (88%), post-operative pain (67%), and mycosis (95%) respectively.

The antidotes and antivenom class were not prescribed by any responders, and emergency (anaphylactic reactions) medications were only occasionally prescribed by Endorsed participants. As expected, there are some medications not recommended by Non-endorsed and In-training participants due to requiring endorsement to prescribe, for example, drugs for gout.

**Fig. 2:**
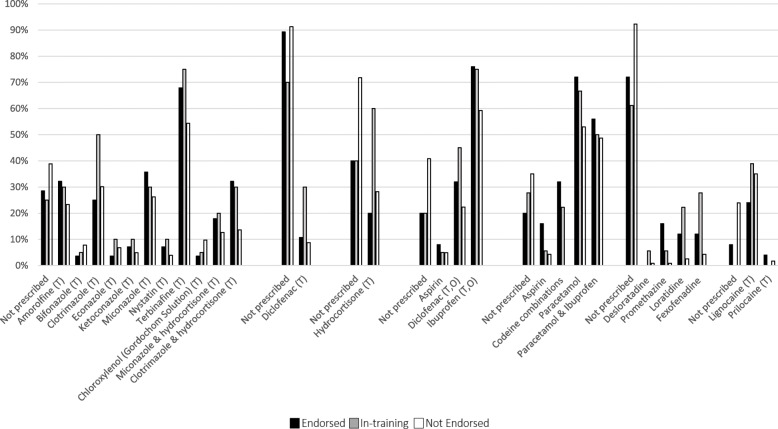
Schedule 2 and 3 medicines, and local anaesthetic prescription/recommendation by podiatrists over the last 12 months, by endorsement status. *Note: topical preparations are denoted by (T) and oral preparations are denoted by (O), In-training = undertaking the process to gain endorsement*

The recommendation of Schedule 2 and 3 medicines by participants over the last 12 months, are presented in Fig. 2 according to endorsement status. A larger proportion of Endorsed participants, or those In-training, reported prescribing/recommending Schedule 2 or 3 medication over the last 12 months compared to Non-endorsed participants. The most prescribed/recommended Schedule 2 medications were ibuprofen, paracetamol, and topical terbinafine, irrespective of endorsement status. The most prescribed Schedule 4 medicines among Endorsed participants included lignocaine (84%), Cephalexin (68%), Flucloxacillin (68%), and Amoxicillin with Clavulanic acid (61%). Lignocaine, a Schedule 4 medicine (local anaesthetic) that podiatrists have long been able to administer without further endorsement, was the most frequently used medication by all participants (84% of Endorsed podiatrists, 75% of podiatrists In-training, 55% of Non-endorsed participants). Except for lignocaine, approximately 1 in 5 Endorsed participants reported ***not*** prescribing medications over the last 12 months. The frequency of prescribing/recommending specific medications (including Schedule 4 medicines) is provided in Supplement Material Table 1.

### Perceptions of the endorsement pathway

#### Facilitators of endorsement

**Fig. 3:**
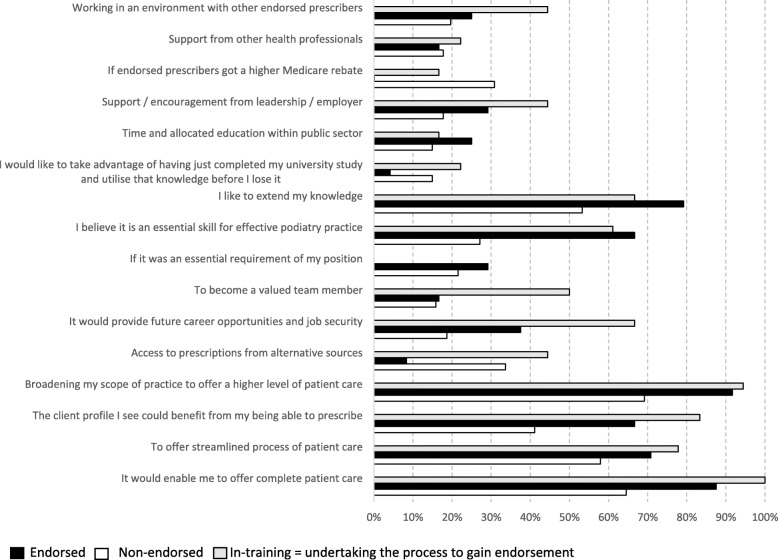
Factors perceived to be important when deciding to undertake endorsement, by endorsement status

The frequency of facilitators for endorsement is provided in Fig. 3. All of the Endorsed participants reported ‘It would enable me to provide complete patient care’, compared with 87.5% of In-training and 64.5% of Non-endorsed participants. Irrespective of endorsement status the items ‘Broadening my scope of practice to offer a higher level of patient care’ was a highly reported facilitator along with ‘To offer streamlined processes of patient care’ and ‘I like to extend my knowledge’.

Compared to Endorsed participants (0.1%), Non-endorsed participants (30.8%) identified ‘If endorsed podiatrists got a higher Medicare rebate’ as an important factor in deciding to undertake the process to gain endorsement. In-training (44.4%) and Non-endorsed (33.6%) participants had similar response rates to ‘Access to prescriptions from alternate sources’, while this item had low response rates from Endorsed participants (8.3%). Compared to Endorsed (66.7%) and In-training participants (61.1%), fewer Non-endorsed participants (27.1%) reported ‘I believe it is an essential skill for effective podiatry practice’.

#### Barriers of endorsement

The results for barriers to endorsement are listed in Table 2. For those that were Endorsed or In-training the most highly reported barriers were ‘Prolonged approval / review process from the Board’ (75.0%), ‘The time commitment involved impacted my private life’ (62.5%), and ‘Time away from work’ (45.8%). Of note, very few (12.5%) Endorsed participants identified that they had ‘No difficulties’ associated with completing the requirements for endorsement.

**Table 2:** Items that respondents thought made endorsement difficult to complete (Endorsed and In-training participants) or acted as barriers to undertaking endorsement (Non-endorsed participants) in rank order

Factors that made it difficult to complete the requirements for endorsement, as reported by endorsed respondents and podiatrists in-training	Endorsed/in-training
Prolonged approval / review process from the Board	75.0%
The time commitment involved impacted my private life	62.5%
Time away from work	45.8%
Rural or remote location offers logistical barriers to access training	20.8%
No tangible incentives to undertake training	20.8%
Limited access to supervisors / mentors	20.8%
Other (please specify)	16.7%
Limited support from supervisors / mentors	12.5%
Not applicable - no difficulties	12.5%
Multiple mentees shadowing one mentor	4.2%
**Barriers to undertaking endorsement, as reported by non-endorsed respondents**	**Non-endorsed**
Limited access to supervisors/ mentors	60.8%
The cost of training is prohibitive - uni or time away from work	53.3%
Lack of structured clinical training	45.8%
Lack of PBS funding	42.1%
I do not have the time needed to undertake training	40.2%
It is harder in private practice/non-hospital-based positions than within the public hospital sector	33.6%
You can be a successful podiatrist without having endorsement	32.7%
No tangible incentives to undertake training	31.8%
Easy and convenient access to prescriptions from alternative sources	29.0%
Lack of understanding of the endorsement training process	21.5%
Lack of professional role identity - Our patients are not aware we can prescribe	20.6%
The profile of patients I treat do not require this service	18.7%
Rural or remote location offers logistical barriers to access training	17.8%
Other (please specify)	16.8%
I am concerned the other health professionals I work with would not approve	10.3%
Staff shortages in rural or remote areas	9.4%
I’m towards the end of my professional career	7.5%
I prefer to use more natural interventions rather than prescription medicines	5.6%
I have just started practicing and would like to get some experience before I undertake endorsement	4.7%
It is harder within the public hospital sector than in private practice/non-hospital-based positions	4.7%

Note: participants could select multiple responses, i.e. all responses that apply

More than half of Non-endorsed participants reported barriers associated with the training process and time requirements. Some of the most highly reported responses were ‘Limited access to supervisors/ mentors’ (60.8%), and ‘The cost of training is prohibitive - University or time away from work’ (42.8%), and ‘Lack of structured clinical training’ (45.8%), and ‘I do not have the time needed to undertake training’ (40.2%). Many Non-endorsed participants reported ‘It is harder in private practice/non-hospital-based positions than within the public hospital sector’ (33.6%). This perception may be because podiatrists in large hospitals have access to a broad range of supervised practice opportunities. ‘Lack of PBS funding for podiatry-prescribed scripts’ (42%), ‘You can be a successful podiatrist without having endorsement’ (32.7%), ‘The easy availability of scripts from alternate sources’ (29.0%), and ‘Lack of professional role identity - Our patients are not aware we can prescribe’ (20.6%) were also identified.

## Discussion

This is the first known survey of Australian podiatrists’ prescribing and recommendation practices, and barriers and facilitators to endorsement for scheduled medicines. Findings from 225 registered podiatrists indicate that approximately one third of survey participants were endorsed podiatrists, well above the percent of endorsed podiatrists (less than 3%) in the Australian podiatry population, which may reflect a greater interest in the survey topic among this group. The most common medications prescribed or recommended by all podiatrists were local anaesthetics, antimycotics, antibacterial agents, and analgesics. These findings are consistent with other studies that examine prescribing and medication use practices among non-medical prescribers [19, 20]. Taken together these findings suggest the management of pain and infection are areas where patients’ patterns of receiving their medications is undergoing change.

Podiatry, with its known association with chronic disease is well placed in the primary health care setting to reduce burden on General Practitioners and the public health care system [4, 10]. These benefits could be maximised if the rate of endorsement is increased in the podiatry profession. While a majority of Non-endorsed participants reported they would like to become an endorsed prescriber, this intention is not converted into behaviour. This intention-behaviour gap could be partly explained by Endorsed participants reported numerous barriers within the process to gain endorsement, with only 12.5% of Endorsed participants reporting no difficulties. Further, 75% indicated that the wait to receive the award after completing training was prolonged. Steps taken by the Podiatry Board of Australia to reduce the wait time to obtain endorsement may address this concern in the future.

The current endorsement Pathway B require a high level of self-motivation. Individuals are tasked with costly enrolment into post-graduate courses (with associated non-Commonwealth supported fees), the commitment to undertake self-directed online case studies, identify and approach a mentor, identify and organise supervised practice opportunities over a broad range of areas, and complete a portfolio of evidence. Hence, it is not unexpected that barriers related to the endorsement process were amongst the most highly reported barriers by Non-endorsed participants. In addition, Non-endorsed participants frequently identified a ‘Lack of understanding of the endorsement training process’. While this could reflect a lack of intent or motivation to engage with the process to become endorsed, it may also reflect the onerous process of completing the requirements to gain endorsement.

The ‘Lack of PBS funding’ was reported as a considerable barrier for Non-endorsed participants to undertake the endorsement process. Eligibility to prescribe medications under the PBS has been viewed as an important factor in providing full episodes of patient care elsewhere [21, 22]. Without PBS subsidies, podiatry patients may incur greater out-of-pocket expenses or choose to return to their General Practitioner for prescriptions, increasing public and healthcare costs.

Many pathology investigations such as blood, urine, or tissue required for the safe and responsible use of scheduled medicines and to provide full episodes of patient care are either not subsided when ordered by podiatrists or podiatrists are unable to request them. Topical antifungal therapies (which are known to have limited efficacy for the treatment of onychomycosis [23]), do not require pathology testing to prescribe and manage and were recommended by up to 67% of Endorsed participants over the last twelve months. While oral terbinafine, which has high quality evidence for efficacy in the treatment of onychomycosis but requires both pathology testing and liver function monitoring [24], was only prescribed by 25% of Endorsed participants over the last twelve months. Such testing can only be accessed by a referral back to a GP, reducing efficiencies. This example demonstrates how limited access to pathology testing may, in-part, explain the prescribing patterns for antifungal medications observed in this study as well as the relatively high proportion of Endorsed participants not prescribing any medications.

In 2018 the estimated total cost to train a podiatrist through the endorsement process in the public hospital system was estimated at over $50,000, inclusive of costs and in-kind costs to the hospital and the podiatrist themselves [25]. Although this appears to be a significant investment, the researchers suggested breakeven could be achieved by averting 0.68 infected diabetic foot ulcers (DFU) from major amputations [25]. DFU is another area of podiatric practice where health system efficiencies and safe prescribing may be further improved by available and/or subsidised pathology testing for podiatrists.

Identifying factors that motivate podiatrists to become endorsed is an important step in developing strategies to increase engagement and participation. The findings of this survey build on the qualitative interviews by Graham et al. [17] confirming that the self-determination theory (SDT) [26, 27] can provide insights into the drivers of motivation. This universal motivational and personality framework is commonly applied in education and health settings and is based on the concept that people naturally develop through acquiring knowledge, skill, and habits they observe that support their basic psychological needs of autonomy, competence, and relatedness [28].

Self-determination theory posits that motivation to undertake learning can be represented on a continuum from extrinsic motivation (e.g. financial rewards) through to intrinsic motivation (e.g. curiosity or finding the content appealing and interesting) [28]. Endorsed participants indicated the intrinsic motivator such as ‘I like to extend my knowledge’ for participating in endorsement training. This may suggest that such intrinsic motivation is more likely to motivate podiatrists to action than the extrinsic factors such ‘Lack of PBS funding’ and ‘No tangible incentives to undertake training’. Interestingly, these were reported as barriers by Non-endorsed participants.

In SDT, internalisation occurs when a behaviour becomes a personally endorsed value such that the behaviour is in harmony with the broader values, commitments, and interests of the person [29, 30]. In-training participants had the highest reporting of ‘I believe it is an essential skill for effective podiatry practice’, followed by endorsed participants, suggesting for them prescribing may have become an internalised behaviour that motivated these participants to overcome the onerous process involved in becoming endorsed.

Several studies have supported aspects of SDT on an organizational level [31]. The history of local anaesthetic (LA) use in podiatry could be one example of internalising prescribing as a behaviour on a profession wide level. Local anaesthetic was introduced as part of undergraduate podiatry training in Australia in 1978 [32] and has seen a wide uptake such that it is now seen by the profession as an essential skill. Findings from this survey support that LA has become an internalised behaviour in podiatry as LA’s were a medication group non-endorsed participants frequently used.

Self-determination theory hypothesises that the need to be connected to others and to be an effective member of the social environment supports the tendency to internalise the values and regulatory processes of their surroundings [31]. In-training participants had the highest reporting of the social and workplace influences of ‘Becoming a valued member of a team’ and ‘Support from employers’. Similarly, In-training participants reported higher levels than Non-endorsed participants of ‘Working in an environment with other endorsed prescribers’, suggesting observing prescribing in the workplace can motivate an individual to master this skill to enable them to better integrate into the larger social structure of the workplace [28]. The lack of internalization by Non-endorsed participants is supported by the reporting of ‘You can be a successful podiatrist without having endorsement’.

### Future directions

The second most frequently reported barrier by Endorsed participants was ‘The time commitment involved impacted my private life’. Training systems that provide flexible learning environments to assist with balancing work and other commitments may overcome this barrier. Plans to incorporate the requirements for endorsement into the undergraduate accredited programs in Australia are one such strategy to assist in increasing endorsement rates in the future podiatry workforce. However, this poses new challenges such as curriculum creep, staffing and resourcing requirements. Forward planning to meet the required mentors for prescription practices in the new undergraduate pathways will maximise rates of participation in endorsement. Supporting podiatrists working in the academic environment to become endorsed could be considered a priority.

This research supports the premise of SDT theory that social context can motivate behaviour change. Combined with the finding that supervisor and mentor access are a major barrier suggests that supporting podiatrists in leadership roles in clinical settings may be a strategy that could have considerable short-term impact by improving access to mentors as well as increase opportunities to internalise prescribing behaviour in the podiatry profession. One strategy could be to establish a formalised leadership and mentoring framework, as well as communities of practice where podiatrists support each other and increase capacity. One example of this style of supportive program being successfully established in podiatry is a mentoring program developed in a Victorian public health service [25].

Podiatry could explore strategies used by other allied health groups included in the legislative changes for non-medical prescribing. For example, in the Australian optometry profession which is comparable in size to podiatry (*N* = 6207), 68% of optometrists hold an endorsement for scheduled medicines qualification [29]. In addition to incorporating therapeutic training into undergraduate degrees since 2002 [30], in optometry there is a streamlined, post graduate academic pathway. A one-year postgraduate certificate in Ocular Therapeutics is offered by two accredited independent optometry education institutions. The cost to students is approximately $10,000 and includes support to meet required clinical hours, such that students graduate with a formalised qualification recognised by their registration body. To the authors knowledge, there are currently 2 universities offering the postgraduate podiatric therapeutic component of the pathway. However, there are currently no Australian providers that offer courses that incorporate all components of the pathway.

The Australian rural and remote population can have greater challenges accessing healthcare than those living in more urban areas. While 10 survey participants reported working in rural areas and one as working in a remote area, none were endorsed. Further research to explore strategies to overcome the significant barriers for this group of podiatrists to undertake the process to become endorsed has the great potential to offer improved equitable and timely access to scheduled medicines in these communities.

### Strengths and limitations

While this survey examines a sample of Australian podiatrists that share demographic characteristics consistent with the Podiatry Board of Australia registrant data, there are limitations that must be addressed. Firstly, the final sample was relatively small, reflecting approximately 4% of the total Australian podiatry population. Caution is therefore needed when generalising study findings to the entire podiatry population. Secondly data collected are self-reported and may be prone to reporting bias such as recall bias and social desirability bias [31]. Lastly, we did not differentiate the separate pathways available to become an endorsed prescriber. Since several endorsement pathways exist, it is possible that perceptions relating to factors that made endorsement training difficult to complete, may vary according to pathway.

## Conclusion

This is the first known research to examine the prescribing and medication recommendation practices of podiatrists within Australia, with a key outcome being that podiatrists predominantly prescribe or recommend medications to assist pain, inflammatory, or infectious conditions. However, lack of PBS funding and pathology testing access limit podiatrists’ ability to provide full episodes of patient care. Therefore, the valuable benefits of streamlined care, improved patient access, and improved efficiencies in the health system may not be fully realised and can even be difficult to examine.

Approaches that improve access to mentors and a broad range of supervised practice, such as a increased numbers of endorsed podiatrists or a formalised leadership and mentoring framework are likely strategies that that could improve rates of endoesement. It was also suggested that some barriers to endorsement could be addressed by internalising prescribing as a behaviour in the podiatry profession through supporting staff within leadership roles and teaching institutions to engage with and model the prescription of scheduled medicines. The plan to incorporate the requirements for endorsement into undergraduate education has been demonstrated in the past to be effective in podiatry when used with the prescription of LA. While this may be a long-term strategy, in the short term, improving access to mentors and incentives for the current workforce to become endorsed should be actively considered.

## Supplementary Information

**Additional file 1.**

## Data Availability

The datasets used and/or analysed during the current study are available from the corresponding author on reasonable request.

## Abbreviations

DFU: diabetic foot ulcers
LA: local anaesthetic
PBS: Pharmaceutical Benefits Scheme
SDT: self-determination theory
