# Supplementary material for: Australian podiatrists scheduled medicine prescribing practices and barriers and facilitators to endorsement: a cross-sectional survey
Source: J Foot Ankle Res. 2022 Feb 8;15:11. doi: 10.1186/s13047-022-00515-w (PMC8822637; doi:10.1186/s13047-022-00515-w)
Supplement: Supplementary file 1 — Additional file 1. [file 13047_2022_515_MOESM1_ESM.docx]

**Supplementary Material**

**Table 1.** “Which of the following medications have you prescribed over the last 12 months”

| Medications | | Endorsed | Non-endorsed | In-training | |
| --- | --- | --- | --- | --- | --- |
| Antimycotics | | n | n | n | |
|  | Not applicable - never prescribed Antimycotic agents over the last 12 months | 8 | 40 | | 5 |
|  | Amorolfine (Topical) - Schedule 2 & 3 | 9 | 24 | | 6 |
|  | Bifonazole (Topical) - Schedule 2 & 3 | 1 | 8 | | 1 |
|  | Clotrimazole (Topical) - Schedule 2 & 3 | 7 | 31 | | 10 |
|  | Econazole (Topical) - Schedule 2 & 3 | 1 | 7 | | 2 |
|  | Ketoconazole (Topical) - Schedule 2 & 3 | 2 | 5 | | 2 |
|  | Miconazole (Topical) - Schedule 2 & 3 | 10 | 27 | | 6 |
|  | Nystatin (Topical) - Schedule 2 & 3 | 2 | 4 | | 2 |
|  | Terbinafine (Topical) - Schedule 2 & 3 | 19 | 56 | | 15 |
|  | Chloroxylenol (Gordochom Solution) (Topical) - Schedule 2 & 3 | 1 | 10 | | 1 |
|  | Miconazole & hydrocortisone (Topical) - Schedule 2 & 3 | 5 | 13 | | 4 |
|  | Clotrimazole & hydrocortisone (Topical) - Schedule 2 & 3 | 9 | 14 | | 6 |
|  | Terbinafine (oral) - Schedule 4 | 7 |  | |  |
|  | Griseofulvin (oral) - Schedule 4 | 0 |  | |  |
| Antibacterials | |  |  |  | |
|  | Not applicable - never prescribed Antibacterials over the last 12 months | 4 |  | |  |
|  | Cephalexin - Schedule 4 | 19 |  | |  |
|  | Clindamycin - Schedule 4 | 12 |  | |  |
|  | Roxithromycin - Schedule 4 | 4 |  | |  |
|  | Metronidazole - Schedule 4 | 4 |  | |  |
|  | Amoxycillin - Schedule 4 | 6 |  | |  |
|  | Amoxicillin with clavulanic acid - Schedule 4 | 17 |  | |  |
|  | Dicloxacillin - Schedule 4 | 12 |  | |  |
|  | Flucloxacillin - Schedule 4 | 19 |  | |  |
|  | Mupirocin - Schedule 4 | 2 |  | |  |
|  | Silver Sulfadiazine - Schedule 4 | 0 |  | |  |
|  | Erythromycin (podiatric surgeons only) - Schedule 4 | 1 |  | |  |
|  | Phenoxymethyl penicillin (podiatric surgeons only) - Schedule 4 | 0 |  | |  |
|  | Ciprofloxacin (podiatric surgeons only) - Schedule 4 | 2 |  | |  |
|  | Doxycycline (podiatric surgeons only) - Schedule 4 | 1 |  | |  |
| Actinic keratoses | |  |  |  | |
|  | Not applicable - never prescribed Actinic keratoses over the last 12 months | 25 | 94 | | 14 |
|  | Diclofenac (topical) - Schedule   2 | 3 | 9 | | 6 |
| Drugs for Gout | |  |  |  | |
|  | Not applicable - never prescribed Antihistamine over the last 12 months | 20 |  | |  |
|  | Colchicine - Schedule   4 | 5 |  | |  |
| Corticosteroids | |  |  |  | |
|  | Not applicable - never prescribed Anti-inflammatories over the last 12 months | 10 | 84 | | 8 |
|  | Hydrocortisone and Hydrocortisone Acetate (Topical) - Schedule 2 & 3 | 5 | 33 | | 12 |
|  | Betamethasone (Injection or Topical) - Schedule 4 | 11 |  | |  |
|  | Dexamethasone (Injection) - Schedule 4 | 7 |  | |  |
|  | Methylprednisolone (Injection or Topical) - Schedule 4 | 5 |  | |  |
|  | Triamcinolone (Injection or Topical) - Schedule 4 | 6 |  | |  |
|  | Hydrocortisone (Topical) - Schedule 4 | 1 |  | |  |
|  | Mometasone (topical) - Schedule 4 | 2 |  | |  |
|  | Desonide (topical) - Schedule 4 | 0 |  | |  |
| Non-steroidal anti-inflammatory | |  |  |  | |
|  | Not applicable - never prescribed Anti-inflammatories over the last 12 months | 5 | 42 | | 4 |
|  | Aspirin - Schedule 2 & 3 | 2 | 5 | | 1 |
|  | Diclofenac (Oral or Topical) - Schedule 2 & 3 | 8 | 23 | | 9 |
|  | Ibuprofen (Oral or Topical) - Schedule 2 & 3 | 19 | 61 | | 15 |
|  | Celecoxib - Schedule 4 | 5 |  | |  |
|  | Indomethacin - Schedule 4 | 4 |  | |  |
|  | Meloxicam - Schedule 4 | 10 |  | |  |
|  | Naproxen - Schedule 4 | 6 |  | |  |
|  | Sulindac - Schedule 4 | 0 |  | |  |
|  | Ketorolac (podiatric surgeons only) - Schedule 4 | 0 |  | |  |
|  | Naproxen- Schedule 2 & 3 |  | 9 | | 5 |
| Analgesia | |  |  |  | |
|  | Not applicable - never prescribed Pain management medications over the last 12 months | 5 | 41 | | 5 |
|  | Aspirin - Schedule 2 & 3 | 4 | 5 | | 1 |
|  | Codeine combinations - Schedule 2 & 3 | 8 | 0 | | 4 |
|  | Paracetamol - Schedule 2 & 3 | 18 | 62 | | 12 |
|  | Paracetamol & ibuprofen - Schedule 2 & 3 | 14 | 57 | | 9 |
|  | Codeine - Schedule 4 | 4 |  | |  |
|  | Oxycodone (podiatric surgeons only) - Schedule 8 | 2 |  | |  |
| Antihistamine | |  |  |  | |
|  | Not applicable - never prescribed Antihistamine over the last 12 months | 18 | 108 | | 11 |
|  | Desloratadine - Schedule 2 & 3 | 0 | 1 | | 1 |
|  | Promethazine - Schedule 2 & 3 | 4 | 1 | | 1 |
|  | Loratidine - Schedule 2 & 3 | 3 | 3 | | 4 |
|  | Fexofenadine - Schedule 2 & 3 | 3 | 5 | | 5 |
|  | Promethazine - Schedule 4 | 2 |  | |  |
| Antidotes & Antivenoms | |  |  |  | |
|  | Not applicable - never prescribed Antihistamine over the last 12 months | 24 |  | |  |
|  | Naloxone - Schedule 4 | 1 |  | |  |
| Local anaesthesia | |  |  |  | |
|  | Not applicable - never prescribed Local anaesthesia over the last 12 months | 2 | 28 | | 0 |
|  | Lignocaine (Topical) - Schedule S2 | 6 | 41 | | 7 |
|  | Prilocaine (Topical) - Schedule S2 | 1 | 2 | | 0 |
|  | Bupivacaine (Plain or with adrenaline) - Schedule S4 | 9 | 2 | | 3 |
|  | Felypressin - Schedule S4 | 0 | 0 | | 0 |
|  | Levobupivacaine (Plain or with adrenaline) - Schedule S4 | 0 | 1 | | 0 |
|  | Lignocaine (Plain or with adrenaline) - Schedule S4 | 21 | 64 | | 15 |
|  | Mepivacaine (Plain or with adrenaline) - Schedule S4 | 4 | 2 | | 0 |
|  | Prilocaine (Plain or with felypressin) - Schedule S4 | 0 | 0 | | 0 |
|  | Ropivacaine - Schedule S4 | 5 | 1 | | 1 |
|  | Procaine - Schedule S4 | 0 | 0 | | 0 |
|  | Amethocaine - Schedule S4 | 0 |  | | 0 |
|  | Methoxyflurane - Schedule S4 | 6 |  | | 0 |
|  | Amylocaine (SA only) - Schedule S4 | 0 |  | | 0 |
|  | Benzocaine (SA only) - Schedule S4 | 0 |  | | 0 |
|  | Butacaine (SA only) - Schedule S4 | 0 |  | | 0 |
|  | Butylaminobenzoate (SA only) - Schedule S4 | 0 |  | | 0 |
|  | Cinchocaine (SA only) - Schedule S4 | 0 |  | | 0 |
|  | Diperodon (SA only) - Schedule S4 | 0 |  | | 0 |
|  | Etidocaine (SA only) - Schedule S4 | 0 |  | | 0 |
|  | Oxybuprocaine (SA only) - Schedule S4 | 0 |  | |  |
| Emergency (anaphylactic reactions) | |  |  |  | |
|  | Not applicable - never prescribed Anaphylactic reactions related medications over the last 12 months | 24 |  | |  |
|  | Adrenaline - Schedule S4 | 1 |  | |  |
| Benzodiazepines | |  |  |  | |
|  | Not applicable - never prescribed Anti-anxiety agents over the last 12 months | 20 |  | |  |
|  | Temazapam - Schedule 4 | 2 |  | |  |
|  | Lorazepam - Schedule 4 | 3 |  | |  |
|  | Diazepam - Schedule 4 | 2 |  | |  |
